# Inflammasome and Mitophagy Connection in Health and Disease

**DOI:** 10.3390/ijms21134714

**Published:** 2020-07-01

**Authors:** Jae-Min Yuk, Prashanta Silwal, Eun-Kyeong Jo

**Affiliations:** 1 Department of Infection Biology, Chungnam National University School of Medicine, Daejeon 35015, Korea; yjaemin0@cnu.ac.kr; 2 Infection Control Convergence Research Center, Chungnam National University School of Medicine, Daejeon 35015, Korea; pst.ktz@gmail.com; 3 Department of Medical Science, Chungnam National University School of Medicine, Daejeon 35015, Korea; 4 Department of Microbiology, Chungnam National University School of Medicine, Daejeon 35015, Korea

**Keywords:** mitophagy, inflammasome, inflammation, infection

## Abstract

The inflammasome is a large intracellular protein complex that activates inflammatory caspase-1 and induces the maturation of interleukin (IL)-1β and IL-18. Mitophagy plays an essential role in the maintenance of mitochondrial homeostasis during stress. Previous studies have indicated compelling evidence of the crosstalk between inflammasome and mitophagy. Mitophagy regulation of the inflammasome, or vice versa, is crucial for various biological functions, such as controlling inflammation and metabolism, immune and anti-tumor responses, and pyroptotic cell death. Uncontrolled regulation of the inflammasome often results in pathological inflammation and pyroptosis, and causes a variety of human diseases, including metabolic and inflammatory diseases, infection, and cancer. Here, we discuss how improved understanding of the interactions between inflammasome and mitophagy can lead to novel therapies against various disease pathologies, and how the inflammasome-mitophagy connection is currently being targeted pharmacologically by diverse agents and small molecules. A deeper understanding of the inflammasome-mitophagy connection will provide new insights into human health and disease through the balance between mitochondrial clearance and pathology.

## 1. Introduction

Inflammasomes are intracellular multiprotein complexes that trigger the activation of the enzymatic activity of caspase-1 to release mature interleukin (IL)-1β and IL-18, and also induce pyroptotic cell death [1,2]. The integrated regulation of inflammasome activation and pyroptosis contributes to promoting the antimicrobial host defense and the clearance of pathogens [3,4,5]. However, aberrant activation of inflammasomes leads to excessive inflammatory and pathological autoimmune responses potentially harmful to the host. The inflammasome-related diseases include metabolic and inflammatory diseases, atherosclerosis, arthritic diseases, gout, tissue injury, infectious diseases, and sepsis [6,7,8].

Autophagy is an intracellular catabolic process for the degradation of large macromolecules and/or damaged organelles. Although autophagy has generally been accepted as a bulk- and nonselective degradation process for intracellular cargoes, numerous studies have highlighted the selective nature of autophagy, targeting various organelles or substances [9]. Mitophagy is a selective autophagy pathway that targets and destroys mitochondria to maintain mitochondrial quality control [10]. Accumulating data suggest that mitophagy processes are mediated through several pathways involving PTEN-induced putative kinase 1 (PINK1)/Parkin and diverse mitophagy receptors under different cell condition contexts [11,12]. Dysregulation of mitophagy leads to mitochondrial dysfunction, increased production of mitochondrial reactive oxygen species (ROS), and translocation of mitochondrial DNA into the cytosol, all of which are involved in the activation of the inflammasome [13,14,15].

Here, we review the crosstalk mechanisms between inflammasomes and mitophagy. Dysregulated inflammasome activation, at least partly due to defective mitophagy, contributes to the pathogenesis of a variety of human diseases [16,17]. The mitophagy pathway has been shown to regulate mitochondrial ROS generation and elimination of damaged mitochondria, thus controlling mitochondrial homeostasis to prevent excessive activation of the inflammasomes [12,18,19]. We also discuss recent progress in identifying various agents and molecules to regulate the mitophagy and inflammasome pathways, and which may provide opportunities for novel therapeutics against a variety of human diseases caused by dysregulation of inflammasome and mitophagy.

## 2. Overview of Inflammasomes

Innate immune responses are the primary immune defense, which is activated by pattern recognition receptors (PRRs) that recognize and respond to pathogen-associated molecular patterns (PAMPs) or damage-associated molecular patterns (DAMPs) [20]. Among PRRs, NOD-like receptors (NLRs) are located in the cytosol and constitute a large family of PRRs that include nucleotide-binding domain, leucine-rich-containing family, pyrin domain-containing-3 (NLRP3) [20]. 

Inflammasomes are crucial signaling platforms for the maturation of IL-1β and IL-18, and pyroptosis through caspase-1-dependent processing [21,22]. The mechanisms and functions of the nucleotide-binding domain leucine-rich repeat-containing receptor (NLR) family and, especially, the NLRP3 inflammasome (i.e., the domain containing pyrin (NLRP)) is the most extensively characterized among numerous NLR family members. The NLRP3 inflammasome comprises NLRP3, the adaptor apoptosis-associated speck-like protein containing a CARD (ASC), and caspase-1, a major polyprotein complex. The absent in melanoma 2 (AIM2) inflammasome is formed by AIM2, ASC, and caspase-1, to recognize double-stranded DNA in the cytosol [23]. Also, NLR family, apoptosis inhibitory protein (NAIP) function as specific cytosolic receptors for several bacterial protein ligands, and they assemble with NLR family CARD domain containing 4 (NLRC4) to form inflammasome which activates caspase-1 [24]. The adaptor protein ASC, a protein comprised of a pyrin domain (PYD) and a CARD, is required for the inflammasome activation of AIM2, NLRP3, and pyrin, all of which carry a PYD domain [25,26,27]. In addition, pyrin (TRIM20), encoded by *MEFV* gene, is a PRR for the pyrin inflammasome in response to bacterial toxins [28,29]. Pyrin inflammasome activation is mediated through effectors that modify RhoA [28].

**Figure 1:**
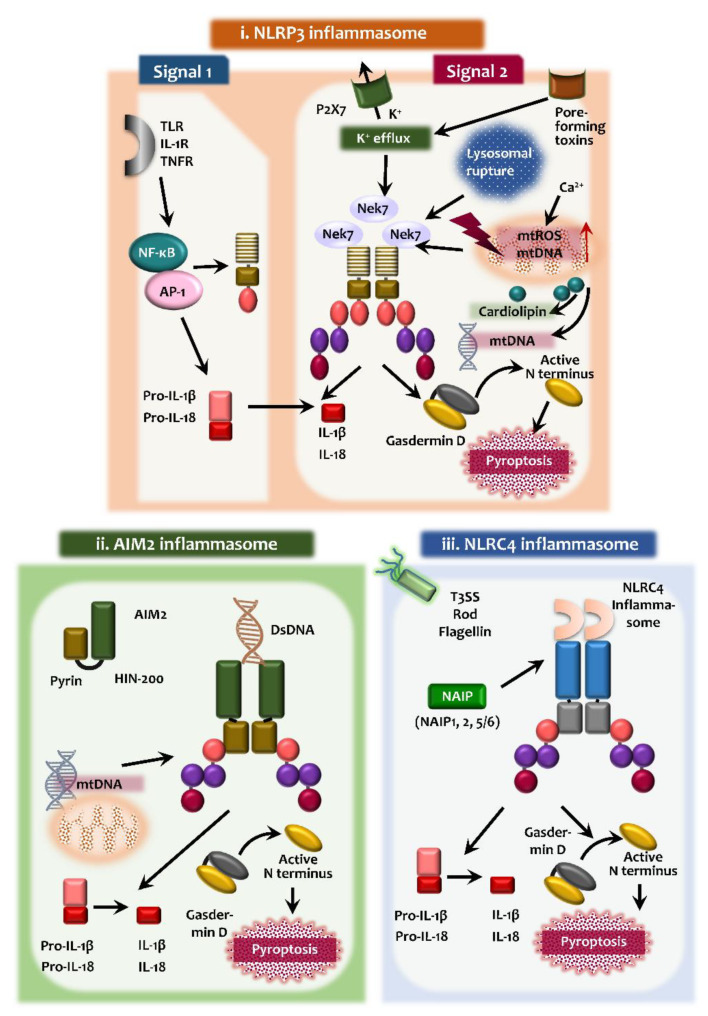
Inflammasome activation. (**i**) A priming signal (signal 1) is necessary for NLRP3 inflammasome activation in response to secondary signal (assembly signal 2). The priming signal through various innate and cytokine receptors such as TLR, IL-1R, and TNFR which activates the transcriptional factor NF-κB to increase the expression of NLRP3 and pro-forms of IL-1β and IL-18. The signal 2 activation by P2X7 receptors, pore forming toxins induce NLRP3 inflammasome assembly and caspase-1 activation leading to processing of pro-forms of IL-1β and IL-18, and the secretion of mature cytokines. Lysosomal destabilization, and mitochondrial damage also induces the NLRP3 activation. A serine-threonine kinase NEK7 facilitates NLRP3 oligomerization. (**ii**) AIM2 inflammasome activation is triggered by sensing the presence of double-stranded DNA. The positively charged surface of the HIN-200 domain binds to the DNA, and the pyrin domain engages to ASC for inflammasome complex assembly. AIM2 can sense the self-DNA and are associated with various pathological conditions. (**iii**) For the activation of NLRC4 inflammasome, NAIP acts as cytosolic receptor for a variety of bacterial protein (T3SS, Flagellin, etc.) and co-assemble into NLRC4 inflammasome complex. All the inflammasome activation lead to pyroptosis through gasdermin D that acts as a key substrate of inflammatory caspases. TLR, Toll-like receptor; IL-1R, interleukin 1 receptor; TNFR, tumor necrosis factor receptor; P2X7, P2X purinoreceptor 7; NEK7, NIMA related kinase 7; T3SS, type III secretion systems. Upward arrow (↑) in red represents increase/induction; all other black arrows represent signaling events.

In this section, we focus on the activation process and roles of the NLRP3, AIM2, and NLRC4 inflammasomes and discuss them in the context of mitophagy, which is summarized in Figure 1.

### 2.1. NLRP3 Inflammasome

Upon pathogen and/or damage-associated stimulation, NLRP3 inflammasome activation is triggered and assembled [30]. The NLRP3 inflammasome is an intracellular multimeric protein complex that is composed of the NLRP3 as the PRR, the adaptor protein ASC, and pro-caspase-1 [30,31]. Although numerous PAMPs and DAMPs are involved in the activation of NLRP3 inflammasome complex, the exact molecular mechanisms have not been fully elucidated to show how NLRP3 inflammasome responds to various PAMPs and DAMPs. It is generally thought that a two-component process, involving innate and cytokine receptor signaling through the Toll-like receptor (TLR) 4, IL-1 receptor (R), and tumor necrosis factor (TNF)-R (priming), and secondary signals (assembly), are involved in NLRP3 inflammasome activation [22,32,33] (Figure 1). 

The first signal for inflammasome priming is essential for the expression of pro-IL-1β and NLRP3 in innate immune cells. Indeed, the inflammasome activation is tightly regulated by a number of factors at the transcriptional level [31]. In addition to NF-κB activation, interferon regulatory factor 1 (IRF1), AP-1, and other transcriptional factors are involved in the transcriptional regulation of the components of NLRP3 inflammasome assembly [31,34,35]. These priming signals also induce post-translational modifications for several inflammasome components, i.e., NLRP3 deubiquitination and the ubiquitination and phosphorylation of ASC, for further activation of inflammasome complex assembly [36]. Although the inflammasome assembly remains fully understood, the second signals are required for the NLRP3 inflammasome assembly. The assembly of NLRP3 inflammasome complexes involves the association of NLRP3 and ASC, and the specks formation of ASC, leading to the recruitment and activation of pro-caspase-1 [36,37]. The second signals include potassium efflux via ATP-dependent P2X purinoreceptor 7 receptor activation, bacterial pore-forming toxins, mitochondrial ROS generation, and lysosomal damage-mediated release of cathepsins such as cathepsin B or L [38]. Pannexin-1, a channel-forming glycoprotein, is an important driver of ATP release during apoptosis, and is also implicated in canonical or non-canonical NLRP3 inflammasome activation during apoptosis [39,40].

Importantly, mitochondrial perturbation and dysfunction are critically involved in NLRP3 activation through enhanced mitochondrial ROS as the second signal [36,41,42,43]. Mitochondrial Ca^2+^ overload leads to the increased generation of mitochondrial ROS, resulting in the activation of the NLRP3 inflammasome [36] (Figure 1). In this event, the mitochondria-associated membrane acts as a platform for inflammasome assembly [41] and cytosolic translocation of mitochondrial DNA act as danger signals for the activation of the NLRP3 inflammasome [44,45]. The molecular mechanisms of upstream signals for NLRP3 activation are not fully understood. Mitochondrial outer membrane proteins such as mitochondrial antiviral signaling protein (MAVS) participate in the NLRP3 inflammasome activation through interaction with NLPR3 [35,46,47,48]. In addition, serine-threonine kinase NIMA related kinase 7 (NEK7) functions as an upstream regulator of NLRP3 inflammasome activation through interactions with- and facilitation of NLRP3 oligomerization [49,50,51].

Pyroptosis is an inflammatory programmed cell death, which is characterized by the release of proinflammatory cytokines (IL-1β and IL-18). To date, two pyroptotic pathways are known; the canonical (caspase-1 mediated) and noncanonical (caspase-4/5/11-mediated) inflammasome pathways [8]. During pyroptosis, gasdermin D (GSDMD) acts as a key substrate of inflammatory caspases (caspase-1/4/5/11). It should be noted that LPS of Gram-negative bacteria can activate a non-canonical inflammasome to induce pyroptosis and the secretion of IL-1β and IL-18 through a direct interaction with human caspase-4 and mouse caspase-11 [52,53]. The GSDMD N-terminal domain has an intrinsic pyroptosis-inducing ability through the formation of cytotoxic pores on the plasma membrane [54,55]. The appropriate activation of the NLRP3 inflammasome and pyroptotic cell death are required for the induction of the innate and adaptive immune responses and antimicrobial host defense against a variety of infections [56,57,58,59]. However, dysregulation of the NLRP3 inflammasome and pyroptosis results in pathological inflammation during infection and is associated with pathogenesis in multiple disease conditions, including inflammatory autoimmune diseases, metabolic, degenerative, and neuroinflammatory diseases [60,61,62]. Therefore, further studies are warranted to understand clearly the complicated mechanisms of NLRP3 activation, which will aid the development of therapeutic agents against NLRP3 inflammasome-associated pathologies.

### 2.2. AIM2 Inflammasome

AIM2 is a cytosolic innate receptor for dsDNA derived from microbes and host cells, and activates ASC-dependent caspase-1, leading to maturation of IL-1β [63,64,65]. AIM2 consists of a PYD domain that associates with ASC, as well as a 200-amino acid repeat (HIN-200) domain that binds the DNA in the cytosol. The positively charged surface of the HIN domain binds to the DNA, and the PYD recruits ASC to assemble the inflammasome complex [64,65,66] (Figure 1). AIM2 inflammasome activation is triggered by infections resulting from DNA viruses and intracellular bacteria, which then participates in the orchestration of the host defense [67,68,69,70]. Since the AIM2 inflammasome can sense self-DNA molecules, its activation is associated with several human diseases, including systemic lupus erythematosus, psoriasis, and tumorigenesis of colorectal cancer [71,72,73]. Circulating cell-free mitochondrial DNA derived from type 2 diabetes patients is able to activate the AIM2 inflammasome in macrophages, suggesting a role for the AIM2 inflammasome in the induction of chronic inflammation during type 2 diabetes [74]. Understanding of the regulatory mechanisms of AIM2 inflammasome activation may offer the potential to prevent various human diseases. 

### 2.3. NAIP/NLRC4 Inflammasome

Among many inflammasomes, NLR family, NAIP/NLRC4 inflammasome is critically involved in the recognition and control of intracellular bacterial infections [24] (Figure 1). The NAIP/NLRC4 inflammasome is the protein complex composed of a single NAIP bound to NLRC4 subunit. The ligands for NAIP/NLRC4 inflammasomes include intracellular bacterial flagellin or the components of type-3 secretion system, derived from pathogens [75]. Once it detects intracellular pathogens, it activates a signaling cascade resulting in pyroptosis and cytokine release to inhibit the replication of pathogenic bacteria [24,76]. At the structural basis, NAIP recognition of bacterial pathogens leads to the oligomerization of NLRC4, to activate the NAIP/NLRC4 inflammasomes that are assembled into multisubunit disk-like structures [24,77,78]. In addition, the gain-of-function mutation of NLRC4 is associated with autoinflammatory diseases in humans [24,79]. Substantial evidence now exists to support a protective role for NAIP/NLRC4 against the infection with *Salmonella typhimurium* in vivo [80], respiratory melioidosis [81]; however, NAIP/NLRC4 inflammasome does not protect against melanoma [82]. Therefore NAIP/NLRC4 inflammasomes are considered to be crucial regulators of infection and immunity.

## 3. Overview of Mitophagy

**Figure 2:**
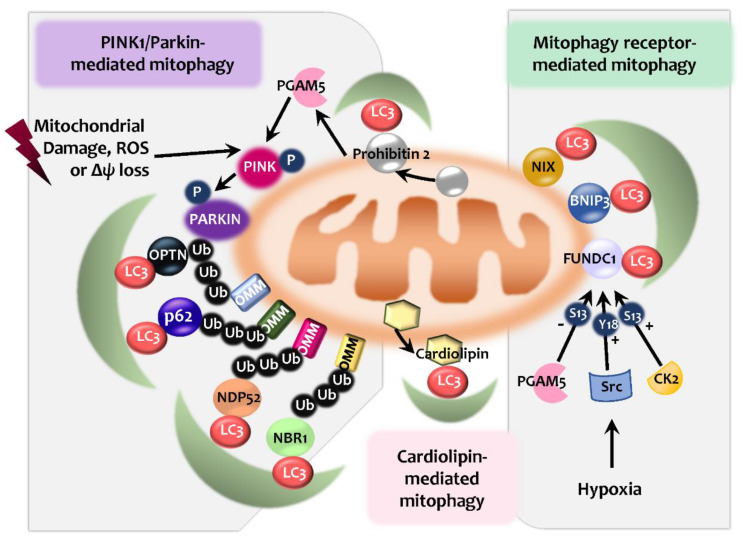
An overview of mitophagy. PINK1/Parkin-mediated mitophagy involves the recognization of dysfunctional mitochondria by PINK1 and activation of Parkin. This leads to ubiquitination of outer mitochondrial membrane-associated proteins, which are then recognized by autophagic receptors such as p62, OPTN, NDP52, and NBR1. These receptors can bridge the cargos with LC3/GABARAP to activate selective autophagy. Inner mitochondrial membrane protein Prohibitin 2 promotes PINK1/Parkin-mediated mitophagy through PGAM5. Mitophagy receptors such as NIX, BNIP3, FUNDC1 present in OMM can interact with LC3 to activate the selective autophagy. FUNDC1 mediates the mitophagy via its dephosphorylation (represented as ‘-‘) at Ser-13 by PGAM5 and phosphorylation (represented as ‘+’) at Ser-13 and Tyr-18 by CK2 and Src kinase respectively. Cardiolipin, by its transmembrane distribution, can activate mitophagy through binding to LC3 or GABARAP. OPTN, optineurin; OMM, outer mitochondrial membrane-associated proteins; GABARAP, γ-aminobutyric acid A receptor-associated protein; PGAM5, PGAM family member 5; CK2, Casein kinase 2; Δψ, mitochondrial membrane potential; ROS, reactive oxygen species; NIX, NIP3-like protein X; FUNDC1, FUN14 domain containing 1.

Mitophagy plays a key role in mitochondrial quality control and homeostasis. It is known that several different effectors participate in the process of mitophagy. These effectors include PINK1/Parkin with autophagic receptors, and multiple mitophagy receptors, such as NIP3-like protein X (NIX; also known as BNIP3L), BNIP3, FUN14 domain containing 1 (FUNDC1), FK506 Binding Protein 8 (FKBP8), and Syntaxin 17 (STX17) [12,83]. Also, Parkin-independent ubiquitin-mediated mitophagy and lipid-mediated mitophagy activation have been discovered [83,84,85,86] (Figure 2). Here, we briefly discuss several mechanisms underlying mitophagy processes, including Parkin/PINK1-induced and mitophagy receptor-mediated pathways.

### 3.1. PINK1/Parkin-Mediated Mitophagy

Mitophagy is a selective autophagic process that primes and targets damaged mitochondria to the lysosomes for degradation [87,88]. PINK1/Parkin-dependent mitophagy is a well-known post-translational signaling cascade that recognizes the cargo through the polyubiquitination of mitochondrial proteins and the recruitment of the autophagic machinery [88,89] (Figure 2). Mitochondrial Ser/Thr kinase PINK1 level is very low through constitutive degradation in physiological condition [90]. Once ΔΨ_M_ is dissipated, translocase of the inner membrane (TIM)23-mediated import of PINK1 is inhibited, thus being accumulated on the outer membrane (OMM) [90]. A supercomplex containing PINK1 homodimers facilitates PINK1 activation to phosphorylate the ubiquitinated substrates on the OMM [90]. In addition, the activated PINK1 recruits and phosphorylates Parkin, the E3 ubiquitin ligase [89,91]. Once recruited, Parkin induces the ubiquitination of the OMM proteins, which can be recognized by several autophagic receptors, including SQSTM1/p62 (sequestosome 1), NBR1, NDP52, and optineurin (OPTN) [87,92,93,94]. The autophagic receptors, having both a ubiquitin-binding domain and LC3-interacting region (LIR) motifs, are able to bridge ubiquitinated cargos with ubiquitin-like proteins belonging to the LC3/ γ-aminobutyric acid A receptor-associated protein (GABARAP) family, and the components of the core autophagic machinery to further activate selective autophagy [87,94,95,96]. Notably, both OPTN and NDP52 may play essential roles in mitophagy, independently of Parkin [97,98], whereas other receptors (p62, NBR1, and TAX1BP1) showed a lesser contribution [92].

In addition, prohibitin 2 (PHB2), containing a LIR domain, is identified as the inner mitochondrial membrane (IMM) mitophagy receptor that works to prime mitochondria during Parkin-dependent mitophagy [99]. PGAM (phosphoglycerate mutase) family member 5 (PGAM5, mitochondrial serine/threonine protein phosphatase) is also required for the participation of PHB2-mediated stabilization of PINK1, and promoting PINK1/Parkin-mediated mitophagy [100] (Figure 2).

### 3.2. The Mitophagy Receptor-Mediated Pathway

In mammals, mitophagy is mediated by specific mitophagy receptors, including NIX, BNIP3, FUNDC1, FKBP8, and STX17. Among these receptors, BNIP3, NIX, and FUNDC1 are the mammalian functional counterparts of yeast Atg32 [101,102] and are recruited to the OMM. As mitophagy receptors, they contain LIRs to enable them to interact with LC3, thereby activating selective autophagy against damaged mitochondria (Figure 2). The BCL2 family proteins BNIP3 and BNIP3L/NIX cooperate with the LC3 family proteins to eliminate damaged mitochondria [92,103]. NIX, a protein on the mitochondrial OMM [104], is essential for erythroid maturation through mitophagy activation in the reticulocytes [105,106]. During viral infection, both NIX/BNIP3L and BNIP3 are required for the generation of natural killer (NK) memory and the enhancement of NK cell proliferation through protective mitophagy activation via the Atg3-dependent mechanism [107]. Additionally, during retinal development, the tissue hypoxia signal induces the expression of NIX/BNIP3L and NIX-mediated mitophagy to induce a metabolic shift toward glycolysis and promote M1/proinflammatory macrophage polarization in retinal ganglion cell development [108]. In addition, FUNDC1 is a crucial mitophagy receptor in hypoxia-induced mitophagy through interaction with LC3 and subsequent activation of mitophagy [109]. FUNDC1-mediated mitophagy is regulated by a phosphorylation/dephosphorylation system, i.e., Unc-51-like autophagy activating kinase (ULK1)-mediated phosphorylation of FUNDC1 at Ser-17 and PGAM5-mediated dephosphorylation at Ser-13 [110,111]. Under normoxic conditions, FUNDC1-mediated mitophagy is inhibited by Src kinase and casein kinase 2 (CK2), which phosphorylate FUNDC1 at Tyr-18 and Ser-13, respectively [103,112].

### 3.3. Lipid-Mediated Mitophagy: Cardiolipin

Cardiolipin, a tetra-acyl anionic phospholipid found at the IMM, is required for the maintenance of mitochondrial membrane structures and normal bioenergetic processes [113,114]. During mitochondrial oxidative stress, cardiolipin is translocated into the external mitochondrial membrane [113,114,115]. Transmembrane redistribution of cardiolipin can lead to the activation of mitophagy through interactions with LC3B or GABARAP [116,117,118] (Figure 2). Mechanistically, the protein kinase C (PKC) pathway [119] and Tafazzin (TAZ), a phospholipid transacylase [120], are involved in the cardiolipin-mediated initiation of mitophagy. However, the regulatory pathway and/or the players involved in the activation of cardiolipin-mediated mitophagy remain largely unknown.

## 4. Mitophagy Crosstalk with the Inflammasome

**Figure 3:**
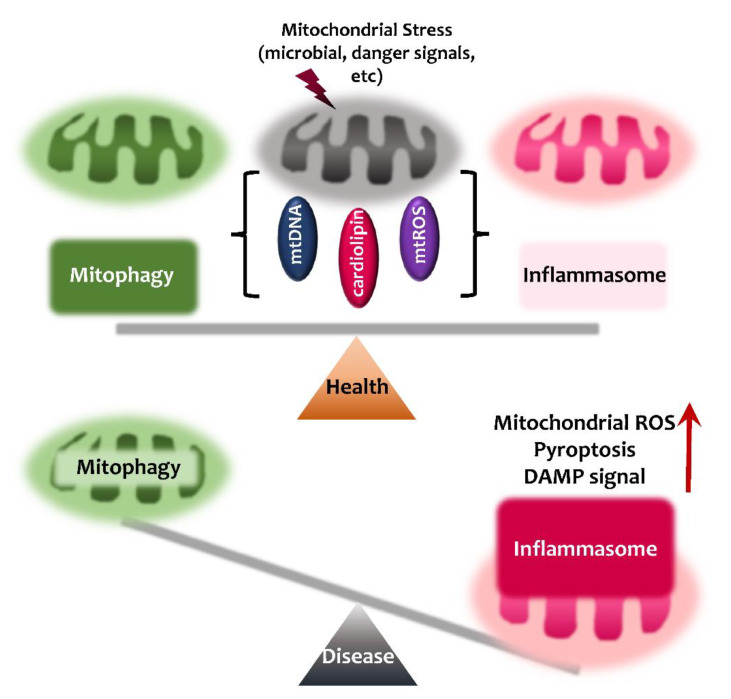
Inflammasome and mitophagy in health and disease. Balance between inflammasome and mitophagy activation is required to prevent the harmful inflammatory responses of microbial or other danger signals, and to maintain protective immunity and good health. Any disproportion between activation of inflammasome and mitophagy causes sustained mitochondrial stress leading to pyroptosis and pathological inflammation.

Although there is increasing evidence of the link between mitophagy and the inflammasome in human health and disease [1,17,121,122,123], few studies have reported the key components through which mitophagy regulates the inflammasome and vice versa. The interrelationship between inflammasome and mitophagy is summarized in Figure 3. The balanced activation of inflammasome-mitophagy pathway may contribute to protective host immunity and the prevention of harmful inflammatory responses, and thus maintain human health. However, dysregulation between inflammasome and mitophagy pathways may lead to pathological inflammatory responses to aggravate mitochondrial damage further, and pyroptotic cell death (Figure 3). In this section, we briefly discuss the molecular mechanisms by which inflammasomes and mitophagy are communicated.

### 4.1. Mitochondrial Reactive Oxygen Species (ROS) Generation and Translocation of Mitochondrial DNA into the Cytosol

As discussed above, perturbation of the mitochondria and the generation of mitochondrial ROS trigger signal 2 for inflammasome activation. Also, the cytosolic translocation of mitochondrial DNA acts as a crucial DAMP signal to activate the NLRP3 and AIM2 inflammasomes [36,74,124,125]. At the same time, mitophagy is activated to remove the damaged mitochondria [88,126]. Defective mitophagy increases the production of mitochondrial ROS and the translocation of mitochondrial DNA into the cytosol, amplifying the increased inflammasome activation [17,43,46,127]. Mechanistically, defective mitophagy leads to mitochondrial dysfunction, impairment of membrane integrity, mtDNA leaking out of damaged mitochondria through opening mitochondrial permeability transition pore (mPTP), forming a vicious cycle that ultimately aggravates insufficient mitochondrial quality control and excessive inflammation [128,129,130]. Furthermore, cardiolipin interacts with NLRP3 [45], can be externalized into the surface of the mitochondria through mitochondrial phospholipid scramblase-3 (PLS-3) [131]. The transfer of cardiolipin from inner to outer membrane of mitochondria is mediated by two inner membrane space proteins, the mitochondrial creatine kinase and nucleoside diphosphate kinase (NDPK-D/NM23-H4) through binding and formation of oligomeric complexes with cardiolipin [131,132,133]. After transport into the outer membrane of mitochondria, cardiolipin can trigger mitophagy and activate the NLRP3 inflammasome complex in a context-dependent manner [131]. However, the mechanisms by which cardiolipin detects mitophagic and inflammasome signaling are not fully understood.

### 4.2. Multiple Regulators That Connect between Inflammasome and Mitophagy

In macrophages, an important innate immune signaling mediator, NF-κB, reportedly adopts the p62-mitophagy pathway as an intrinsic system for tissue repair and anti-inflammatory homeostasis. The NF-κB-p62-mitophagy pathway is required to control excessive activation of the NLRP3 inflammasome and IL-1β-mediated inflammation [134]. IL-10, an anti-inflammatory cytokine, inhibits the mammalian target of rapamycin (mTOR) activity, promotes mitophagy, and eliminates dysfunctional mitochondria in macrophages. IL-10 signaling is protective against colitis through the regulation of the NLRP3 inflammasome and IL-1β secretion. Also, the oxidation of phospholipids, such as oxidized phosphatidylcholine, during cellular stress and damage, triggered the activation of the NLRP3 inflammasome and secretion of IL-1β in macrophages [135]. Moreover, deficiency of the kinase JNK2 leads to defective mitophagy, tissue damage, and hyperactivation of inflammasomes in a mouse sepsis model [136], suggesting a role for JNK2 in the regulation of stress-induced mitophagy. These findings suggest that innate immune players participate in the connection between inflammasome and mitophagy to maintain immunological homeostasis. 

In addition, the autophagy protein, GABARAP has been found to regulate NLRP3 inflammasome activation through the clearance of damaged mitochondria in macrophages [137]. Although this study suggests that GABARAP functions in mitochondrial quality control in macrophages [137], the mechanisms by which GABARAP regulates mitophagy remain to be determined. Another study showed that the metabolic component choline transporter CTL1 links mitophagy and the inflammasome. Blockade of choline transporter CTL1 expression or choline phosphorylation inhibits NLRP3 inflammasome activation through the activation of AMPK and the subsequent stimulation of mitophagy [138]. These data strongly suggest that choline kinase inhibitors are beneficial in attenuating acute and chronic inflammation driven by IL-1β.

Interestingly, specific cell types participate in the regulation of the inflammasome activation of other cell types. A previous study has shown that mesenchymal stromal cells have beneficial effects on systemic inflammation during sepsis through mitophagy activation and suppression of mitochondrial ROS and the NLRP3 inflammasome in macrophages [139]. However, it is unclear whether cell-to-cell interaction or soluble factors regulated the inflammasome/mitophagy axis.

## 5. Crosstalk between Inflammasomes and Mitophagy during Infection

Appropriate regulation of the inflammasome pathways is crucial for the activation of the host defense against a variety of microbial infections. However, dysregulation of NLRP3 inflammasome activation can lead to pathological responses during infection. Emerging data suggest a role for mitophagy in various bacterial, viral, and parasitic infections [140]. We discuss the current understanding of the interaction between inflammasome and mitophagy pathways in the context of various infections.

### 5.1. The Inflammasome-Mitophagy Axis in Bacterial Infections

*Pseudomonas aeruginosa* (PA) activates the NLRC4 inflammasome via T3SS-dependent mitochondrial damage and ROS [141]. Mitophagy activation to remove damaged mitochondria markedly attenuated PA-induced NLRC4 inflammasome activation [141]. Several studies have reported the virulence mechanism by which bacteria escape from inflammasome activation. The bacterial Type I CRISPR-Cas system of the PA strain UCBPP-PA14 inhibits TLR4 recognition by targeting the mRNA of LasR, thus diminishing the proinflammatory responses of the host cell and in in vivo models [142]. Mechanistically, the bacterial Type I CRISPR-Cas system of PA activates mitophagy to promote bacterial escape ability from the host immunity through TLR4-mediated inflammation and inhibition of inflammasome activation [143]. MyD88-dependent inflammasome activation and defective autophagy/mitophagy contributed to the pathogenesis of acute liver injury induced by *Ehrlichia*, a gram-negative bacteria [144].

In addition, *Lactobacillus johnsonii* L531 ameliorated Salmonella-induced enteritis through suppression of mitochondrial damage and regulation of NLRC4 and NLRP3 inflammasome activation. Although the mechanism is unclear, *L. johnsonii* L531 played a role in the attenuation of impaired mitophagy in the host cells during *Salmonella infantis* infection [145]. Through the elimination of damaged mitochondria *L. johnsonii* L531 ameliorates enteritis in a *S. infantis* model [145]. Together, these findings suggest that bacterial pathogens modulate the inflammasome/mitophagy axis, which in turn regulates host defensive pathways during bacterial infections.

### 5.2. The Inflammasome-Mitophagy Axis in Viral Infections

During influenza A virus infection, RIPK2 regulates mitophagy through phosphorylation of ULK1, which is needed to control mitochondrial ROS and the activation of caspase-1. NOD2-RIPK2 signaling has been found to be protective against influenza viral infection and immunopathology through inhibition of the NLRP3 inflammasome via ULK1-dependent mitophagy [146]. 

In human astrocytes, HIV infection induces mitochondrial injury and inflammasome activation, which contributes to the development of neurodegenerative diseases [147]. In astrocytes that were productively infected with HIV, mitophagy activation was required for cell death resistance through the inhibition of mitochondrial ROS and injury. In bystander cells with abortive infection, impaired mitophagy resulted in excessive mitochondrial damage and inflammasome-induced cell death [147]. These data suggest that crosstalk between mitophagy and the inflammasome determines the cell fate in the context of productive and abortive HIV infection [147]. Additionally, specific GU-rich single-stranded RNA from the HIV long terminal repeat region (ssRNA40) has been shown to be capable of triggering NLRP3 inflammasome activation in glial cells and cause neurotoxicity [148]. Also, ssRNA40 mediated the impairment of autophagy/mitophagy and was not able to control NLRP3 inflammasome activity and the secretion of neurotoxic cytokines [148]. Furthermore, the HIV-1 transactivator of transcription (TAT) protein increased the expression of mitophagy signaling proteins, such as PINK1, PRKN, and DNM1L, but decreased mitophagy flux, leading to the enhancement of proinflammatory cytokines, such as *Tnf*, *Il1b*, and *Il6* [149]. Other studies showed that either the HIV TAT protein or the HIV-1 viral protein R (Vpr) induces the priming signal (signal 1) to activate the NLRP3 inflammasome complex and the secretion of IL-1β in macrophages and microglia, thus promoting neuroinflammation during HIV infection [150,151].

Parkin appeared to be a critical regulator of the antiviral response through inhibition of antiviral inflammation [152]. Parkin deficiency promotes antiviral immune responses and improves viral clearance and survival by enhancing the mitochondrial ROS-mediated NLRP3 inflammasome pathway [152]. Also, Parkin expression levels are downregulated in peripheral blood mononuclear cells from patients during viral infection [152]. Together, these studies provide new insights into the regulatory functions of mitophagy during the host defense against viral infections.

## 6. Altered Crosstalk between Inflammasome and Mitophagy in the Context of Human Disease

Defective inflammasome and mitophagy activation contribute to the pathogenic responses in a variety of inflammatory and degenerative diseases. In this session, we will briefly introduce several examples for human diseases related with dysregulated crosstalk between inflammasome and mitophagy, prior to the discussion of pharmacologic modulation in the following session. 

### 6.1. Metabolic and Cardiovascular Diseases

Human type 2 diabetes (T2DM) is one of the best characterized human diseases associated with defective autophagy and NLRP3 inflammasome activation. Palmitate (PA) induces defective mitophagy, increased mitochondrial ROS production, and NLRP3 inflammasome activation, thus aggravating lipotoxicity and leading to insulin resistance [153]. In T2DM mice, the impairment of mitophagy and activation of inflammasomes AIM2 and NLRC4 in cardiomyocytes and cardiac macrophages are associated with myocardial infarction [154]. On the other hand, upregulation of autophagy and dysregulation of inflammation may contribute to detrimental effects in cardiac pathology, resulting in the decline of cardiovascular function and heart failure [155,156]. 

Acute exercise strongly triggers mitochondrial ROS-mediated NLRP3 inflammasome activation and the induction of mitophagy proteins, including Beclin1, LC3, BNIP3, and NLRP3 in the rat myocardium [157]. Although the relationship between the inflammasome and mitophagy was not examined, the data suggest that elevated mitophagy minimizes inflammation-induced myocardial injury [157]. These data also suggest that the maintenance of mitochondrial homeostasis by controlled activation of the inflammasome/mitophagy axis could provide a new preventive and therapeutic platform against cardiovascular disease, including heart failure and myocardial infarction associated with T2DM.

In the context of cardiovascular diseases, dysregulation of the cholesterol pathway leads to mitochondrial dysfunction and ROS production, which are related to defective autophagy/mitophagy and further activation of the NLRP3 inflammasome [122]. Lipid-activated eukaryotic initiation factor 2 α (eIF2α) signaling suppresses Parkin-mediated mitophagy, and reduces mitochondrial damage and inflammasome activation [158]. Since eIF2α phosphorylation is persistently activated in mouse and human atheroma, blockade of the eIF2α-mediated stress response could prove useful in the treatment of atherosclerosis [158]. In addition, development of nonalcoholic steatohepatitis (NASH) is linked to the impaired mitophagy leading to hepatic NLRP3 inflammasome activation [159]. These data suggest that the pathogenesis of metabolic and cardiovascular diseases is likely to be associated with defective mitophagy/autophagy and promotion of the inflammasomes.

### 6.2. Neuronal and Nephrotic Inflammation, and Sepsis

Defective mitophagy, mitochondrial dysfunction, and dysregulated activation of inflammasomes are associated with pathogenesis of neurodegenerative diseases such as Alzheimer’s disease, Parkinson’s disease, and multiple sclerosis [160,161]. Systemic inflammation can activate TLR4 and NLRP3 inflammasome in the microglial cells, leading to neuroinflammation, accumulation of fibrillary amyloid-β, and neurodegeneration [162]. In chronic cerebral hypoperfusion models, microglial cells are excessively activated, and ROS are accumulated, in combination with the activated NLRP3 inflammasome and IL-1β production [163]. The inflammasome activation occurs in parallel with mitophagy in another model of retinal neurodegenerative disease, in which pathogenesis was associated with NLRP3 inflammasome activation [164]. 

Several factors involved in the mitophagy process are shown to regulate nephrotic, intestinal, and systemic inflammation. In a contrast-induced acute kidney injury (CI-AKI), the activation of PINK1-Parkin-mediated mitophagy ameliorates tissue damage and suppress apoptosis in renal tubular epithelial cells (RTECs) through the inhibition of mitochondrial ROS and NLRP3 inflammasome activation [165]. In addition, the role of PHB2 was reported in the context of inflammasome/mitophagy axis in a model of renal tubular injury. Silencing of PHB2 inhibits mitophagy and increases renal cell death, whereas PHB2 overexpression suppresses NLRP3 inflammasome activation in renal proximal tubular cells by attenuating mitochondrial dysfunction [166]. In renal biopsy tissue samples from patients with diabetic nephropathy, decreased renal expression of OPTN is negatively associated with urinary levels of IL-1β and IL-18. Also, the treatment of RTECs leads to a significant decrease in OPTN mRNA and protein levels. However, RTECs overexpressing OPTN enhance mitophagy, which decreased NLRP3 activation and the release of IL-1β and IL-18 [161]. These data collectively suggest that the autophagic receptors PHB2 and OPTN are clinically relevant in renal injury/inflammation through inhibition of the NLRP3 inflammasome by mitophagy activation. 

Previous studies showed that *pink1^-/-^* and *park2*^-/-^ mice have an increased susceptibility to polymicrobial sepsis through NLRP3-dependent inflammasome activation [167]. In septic patients, NLRP3 levels are upregulated, whereas PINK1 and PARK2 levels are downregulated, in peripheral blood mononuclear cells [167]. These data indicate that targeting mitophagy factors may provide new therapeutics against neuronal and nephrotic inflammation/injury and sepsis through the inhibition of NLRP3 inflammasome activation. 

### 6.3. Cancers

Mitophagy is induced by a variety of stresses, including hypoxia, nutrient starvation, and mitochondrial and DNA damage, which are all present during tumorigenesis. Thus, emerging evidence suggests roles for mitophagy in the crosstalk with the inflammation and inflammasome pathways in the context of cancer [168]. In a pancreatic cancer mouse model, deficiency of *Pink1* and *Park2* accelerated pancreatic tumorigenesis through mitochondrial iron accumulation and AIM2 inflammasome activation in tumor cells [169]. Genetic depletion of AIM2 in *pink1^-/-^* and *park2*^-/-^ mice increases the protective effects in pancreatic tumorigenesis [169]. In addition, a low level of PARK2, but increased AIM2 expression, has been associated with a poor prognosis in pancreatic cancer patients [169]. FUNDC1, a mitophagy receptor, was accumulated in human hepatocellular carcinomas (HCCs), and played a protective role in the initiation and development of HCCs [170]. In FUNDC1-depleted hepatocytes, dysfunctional mitochondria were accumulated, leading to activation of the inflammasome, excessive production of IL-1β, and hyperproliferation [170]. Thus, defective mitophagy-mediated dysregulation of mitochondrial homeostasis and the resulting activation of the inflammasome may contribute to tumorigenesis, and could be a potential target for therapeutic intervention. 

## 7. The Pharmacological Regulation of Inflammasome-Mitophagy Axis to Control Human Disease

Emerging studies have revealed multiple agents/small molecules as potential therapeutic strategies based on the connection between inflammasome and mitophagy for the treatment of various human diseases. In this section, we discuss the current knowledge on pharmacological regulation of crosstalk between inflammasomes and mitophagy and the potential mechanisms by which the agents/molecules regulate the pathological responses during disease, which are summarized in Table 1.

### 7.1. Pharmacological Regulation in Models of Metabolic and Cardiovascular Diseases

In T2DM patients, metformin therapy increases the expression of mRNA and proteins of mitophagy markers and p-AMPKα (T172), and improve mitochondrial morphology in mononuclear cells from a metformin-treated patient group [171]. The expression levels of mitophagy kinase, PINK1, significantly correlate with homeostatic model assessment of β-cell function (HOMA-β) indices, which represent the insulin sensitivity [171]. Another drug, Miltefosine, an FDA-approved drug for the treatment of leishmaniasis, confers beneficial effects on lipid homeostasis through the activation of lipophagy and mitophagy, autophagy-inducing AMPK, and ULK1 in macrophages [172]. Additionally, Miltefosine inhibits TLR signaling and NLRP3 inflammasome assembly/activity, thereby affecting both signals 1 and 2 of NLRP3 inflammasome activation, and IL-1β release [172]. 

The protective effects in NASH conferred by liraglutide, a glucagon-like peptide-1 (GLP-1) analog, are mediated through the inhibition of the NLRP3 inflammasome and pyroptosis, and activation of mitophagy in hepatocytes. Liraglutide treatment reduces lipid accumulation, inhibits NLRP3 inflammasome and pyroptosis activation, attenuates mitochondrial dysfunction and ROS generation, and augments mitophagy in hepatocytes [175]. Another study using a NASH model reported that a high-fat diet or treatment with palmitic acid leads to impaired mitophagy, contributing to the activation of the hepatic NLRP3 inflammasome [159]. Several pharmacological inhibitors for the NLRP3 inflammasome (MCC950, N-acetyl cysteine or acetyl-L-carnitine) recover mitophagic flux, at least partially, and inhibit inflammasome activation and pyroptosis, thus being helpful in improving the NASH model [159].

### 7.2. Pharmacological Regulation in Models of Neurological and Nephrotic Diseases

The fatty acid amide hydrolase inhibitor URB597 (URB) [183] reduced the impairment of mitophagy and inhibition of the NLRP3 inflammasome pathway [163]. The beneficial effects of melatonin are reported in a subarachnoid hemorrhage model, by activation of mitophagy and inhibition of the NLRP3 inflammasome [174]. Based on the well-established effect of inflammation and mitophagy in traumatic brain injury, simultaneous activation of mitophagy and blockade of the NLRP3 inflammasome is achieved by combined treatment with MCC950 (an NLRP3 selective inhibitor) and Rapamycin (an mTOR inhibitor) [184]. In addition, ATF4 overexpression reduces cerebral ischemia/reperfusion (I/R) injury through enhancement of Parkin expression, mitophagy activity, and inhibition of NLRP3 inflammasome activation [185]. Importantly, mitophagy inhibition by either mdivi-1, an inhibitor of mitophagy, or Parkin knockdown, significantly counteracts the effects of ATF4 to inhibit NLRP3 inflammasome activation [185]. In Alzheimer’s disease model, 2,3,5,4’-Tetrahydroxystilbene-2-O-β-D-glucoside (TSG), the major active component extracted from *Polygonum multiflorum*, reduces β-amyloid-mediated inflammation through AMPK/PINK1/Parkin-dependent mitophagy activation [160]. Given these results, autophagy/mitophagy, combined with NLRP3 inflammasome suppression, may contribute to the development of new strategies for neuroprotection in neurodegenerative and neurological inflammation or injury [184].

In addition, auranofin, an inhibitor of the thioredoxin receptor (TrxR), leads to mitochondrial dysfunction (reduced Δψm and ATP), oxidative stress (increased H_2_O_2_), and mitophagy activation in a human retinal pigment epithelium (RPE) cell line (APRE-19) [186]. Auranofin increases NLRP3 inflammasome activation, which is improved by the antioxidant N-acetylcysteine, mitochondrial antioxidant, and anti-inflammatory drugs (amlexanox and tranilast) [186]. Moreover, mitochondrial protection by SS-31 (elamipretide) contributes to the reduction of mitochondrial damage and inflammasome activation during acute kidney injury (AKI) and arrests the progression of chronic kidney disease (CKD) [176]. Together, these data suggest that multiple agents/molecules targeting inflammasome-mitophagy/autophagy axis are promising candidates for future therapeutics against neurodegenerative diseases and renal injury/inflammation.

### 7.3. Pharmacological Regulation in Models of Inflammatory Disease: Sepsis and Colitis

Either a dopamine agonist (e.g., pramipexole) or an NLRP3-inhibitor (e.g., MCC950) suppresses the sepsis-mediated lethality in *pink1^-/-^* and *park2*^-/-^ mice, suggesting that PINK1-PARK2-mediated neuroimmunology circuits are involved in the protection against septic shock [167]. Also, SESN2 (sestrin 2), a stress-inducible protein, functions in the inhibition of NLRP3 inflammasome activation through the induction of mitophagy [177]. Sesn2-deficient mice show defective mitophagy and increase the susceptibility to sepsis [177].

In an ulcerative colitis (UC) model, palmatine, a herb-derived isoquinoline alkaloid, has protective effects on dextran sulfate sodium (DSS)-induced colitis by enhancing mitophagy, which inhibits NLRP3 inflammasome activation [178]. In the colonic tissue of DSS mice and THP-1 macrophages, palmatine treatment enhances the expression of mitophagy-related proteins, including LC3, PINK1, and Parkin [178]. In addition, ginsenoside Rd alleviates DSS-induced colitis pathologies through autophagy/mitophagy-mediated inhibition of the NLRP3 inflammasome [179]. Andrographolide (Andro), a small molecule, protects mice against colitis-induced colon carcinogenesis by activating mitophagy, suppressing the NLRP3 inflammasome, and reducing IL1B secretion [180]. Further studies are warranted to gather evidence of future therapeutic molecules/agents based on increased mitophagy and suppression of inflammasome activation in the context of sepsis, colitis, or colitis-mediated carcinogenesis.

### 7.4. Pharmacological Regulation Targeting Sirtuins Nuclear Receptor Rev-erbα

Resveratrol, an agent to activate sirtuins 1 and 3, induces autophagy/mitophagy through the activation of AMPK in peritoneal mesothelial cells to ameliorate inflammatory responses triggered by mitochondrial ROS-mediated NLRP3 inflammasome activation [181]. Furthermore, honokiol (HNK), an activator of sirtuin 3 [187], attenuates mitochondrial damage and ROS production, and increases the levels of autophagy/mitophagy markers, including Parkin and PINK1 after surgery/sevoflurane treatment. Importantly, HNK-mediated mitophagy suppresses NLRP3 inflammasome activation and neuroinflammation, which is reversed by the inhibition of an autophagy inhibitor [182]. These data suggest that HNK-induced mitophagy exhibits neuroprotective effects through the regulation of NLRP3 inflammasome activation, thus suggesting its potential use in neurodegenerative diseases. 

Melatonin treatment shows a preventive effect on the progression of atherosclerosis by the inhibition of NLRP3 inflammasome activation through scavenging ROS via mitophagy activation in macrophages [173]. Mechanistically, the melatonin-mediated protective effect is counteracted by knockdown of Sirt3 or pharmacologic inhibition of autophagy, suggesting a role for Sirt3-mediated mitophagy [173]. In addition, the nuclear receptor Rev-erbα (NR1D1) controls autophagy and mitochondrial biogenesis [188] and is associated with circadian variation of NLRP3 [189,190]. Rev-erbα activation inhibits fulminant hepatitis, colitis, Myocardial I/R, and lung infection in an NLRP3 dependent manner [189,190,191,192,193]. Because melatonin and sleep are controlled by the circadian clock, targeting Rev-erbα may give us more insights into the regulation of mitophagy/autophagy-NLRP3 inflammasome axis in these contexts. Future studies are warranted to investigate the possibility of linking sirtuins and/or nuclear receptor Rev-erbα in the activation of mitophagy through amelioration of numerous pathological responses induced by the NLRP3 inflammasome.

## 8. Conclusions

Multiple stimulants involved in the activation of the inflammasome are capable of triggering mitochondrial dysfunction, which displays a depolarized membrane potential, and is therefore targeted for mitophagy and further elimination [12,18,19]. Furthermore, by eliminating the damaged mitochondria, mitophagy controls the activation of the inflammasome to prevent tissue damage [16,17]. Accumulating evidence has increased our knowledge of a variety of biological and pathological events that are induced by crosstalk between inflammasomes and mitophagy. However, the mechanisms underlying this relationship between inflammasomes and mitophagy pathways remain largely unknown. Some additional questions remain to be answered: Are there specific molecules/factors that regulate the balance of inflammasomes and mitophagy to maintain intracellular homeostasis? Can specific types of mitophagy or mitophagy receptors play different roles in regulating inflammasomes and pyroptotic cell death? If specific types of mitophagy and/or mitophagy receptors have a unique role, which molecular targets are required to regulate inflammasome activation? 

Dysregulated mitophagy is linked to aberrant activation of the inflammasome pathway and results in a variety of human diseases, including inflammatory, neurodegenerative, and cardiovascular diseases. Several experimental studies have identified numerous pharmacological agents and/or small molecules for control of the multilayered and bidirectional interactions between inflammasomes and mitophagy. However, the clinical significance of pharmacological regulation of the inflammasome/mitophagy connection is largely unknown in the context of human diseases. Future studies on the crosstalk between inflammasomes and mitophagy pathways will move the field forward and aid our understanding of how mitochondrial function interacts with host innate immunity in human health. Understanding the mechanisms underlying the dysregulation of inflammasomes and mitophagy could provide protective and therapeutic targets against numerous human diseases and pathological conditions.

## Figures and Tables

**Table 1:** Therapeutic candidates based on the regulation of mitophagy-inflammasome connection in human diseases.

Agents/Drugs	Disease/Model	Functions	Underlying Mechanisms	Experimental Objects	Reference
**Metformin**	Type 2 diabetes	↓ Mitochondrial oxidative stress and distortions in mitochondrial morphology	↑ Protein expression of p-AMPKα (T172), and NLRP3 ↑ Protein expression of mitophagy-related markers (PINK1, PARKIN, MFN2, NIX, LC3-II, LAMP2)	Patient samples, PBMCs	[171]
**Miltefosine**	Atherosclerosis	↑ ABCA1-mediated cholesterol release ↓ LPS-mediated NLRP3 inflammasome assembly and IL-1β release ↑ Mitophagy induction	↑ Disruption of lipid-rafts ↑ Inhibition of phosphatidylserine flip ↓ LPS-mediated mitochondrial ROS generation ↓ Loss of mitochondrial membrane potential in murine macrophages treated with LPS	HEK293-ABCA1-GFP cells, BHK cells, RAW264.7, BMDM	[172]
**Melatonin**	Atherosclerosis	↓ Atherosclerotic plaque progression and serum IL-1β level in vivo murine models ↓ NLRP3 inflammasome activation in atherosclerotic lesions and in Ox-LDL-treated murine macrophages	↑ Sirt3-dependent mitophagy induction and reactive oxygen species (ROS) scavenging ↑ Parkin-mediated mitophagy process through Sirt3-FOXO3a pathway	RAW264.7, *ApoE^−/−^* mice	[173]
	Subarachnoid hemorrhage	↓ Brain edema and neurological dysfunction in rat receiving SAH ↓ SAH-induced neuronal cell death in the ipsilateral basal cortex ↓ Pathological changes in mitochondria	↑ Protein expression of autophagy markers (LC3-II/LC3-I and Atg5) and mitophagy markers (Parkin and PINK-1) ↓ ROS and pro-inflammatory cytokines generation ↓ NLRP3 inflammasome activation	Endovascular perforation-induced SAH rat model, Brain tissue	[174]
**Liraglutide**	Non-alcoholic steatohepatitis	↓ Lipid accumulation in hepatocytes ↓ NLRP3 inflammasome-induced hepatocyte pyroptosis ↑ Mitophagy induction	↓ Mitochondrial dysfunction and reactive oxygen species generation ↑ Co-localization of PINK1/Parkin and mitochondria and OPTN and NIX expressions in HepG2 cells treated with LPS and PA	HepG2 cell line	[175]
**URB597**	Chronic cerebral hypoperfusion	↓ CCH-induced NLRP3 inflammasome activation, impaired autophagy, and defective mitophagy in rat hippocampus	↑ The restoration of lysosomal function ↓ CCH-induced microglial overactivation ↓ CCH-induced ROS accumulation	BCCAo-induced CCH rat model, Hippocampus	[163]
**Elamipretide (SS-31)**	Bilateral renal ischemia	↓ Ischemia-induced tubulointerstitial fibrosis and glomerulosclerosis ↓ Inflammatory Response and endothelial Injury after Ischemia	↓ NLRP3 inflammasome activation and upregulation of IL-18, IL-1β, and TNF induced by ischemia ↑ Mitochondrial integrity and autophagic vacuoles containing organelles and cytoplasmic contents in podocytes	Ischemia-reperfusion experimental rat model, Kidney tissue	[176]
**SESN2**	Sepsis	↓ Clearance of damaged mitochondria in *sesn2*^−/−^ macrophages ↑ IL1β and IL18 production in serum and lung tissue of *sesn2*^−/−^ mice after CLP ↑ Mortality rate in *sesn2*^−/−^ mice	↑ Mitochondrial priming by mediating aggregation of SQSTM1 and its binding to lysine 63-linked ubiquitins on the mitochondrial surface ↑ Activation of autophagic machinery for the degradation of primed mitochondria via an increase of ULK1 protein ↓ Prolonged NLRP3 inflammasome activation by inducing mitophagy	*sesn2*^−/−^ mice, *sesn2*^−/−^ GFPM -AP1-LC3 mice, *nos2*^−/−^ mice, *nlrp3*^−/−^ mice, BMDM	[177]
**Palmatine**	Ulcerative colitis	↓ Pathological and histological scores in DSS mice · ↓ Colonic inflammation in DSS mice	↓ NLRP3 inflammasomes activation in DSS mice and THP-1 ↑ PINK1 and Parkin-driven mitophagy in DSS mice and THP-1	DSS-induced UC model using C57BL/6 mice, THP-1 cell	[178]
**Ginsenoside Rd**	Ulcerative colitis	↓ Body weight loss, stool consistency alterations and blood loss in DSS mice ↓ Recruitment of inflammatory cell into colonic tissue ↓ NLRP3 inflammasome activation in colon tissue and THP-1 cells	↓ LPS plus ATP-induced mitochondrial perturbation and mtDNA release in THP-1 ↑ p62 expression and its translocation to mitochondria ↑ p62-mediated autophagy activation ↑ AMPK/ULK1signaling pathway in vitro and in vivo	DSS-induced UC model using BALB/C mice, THP-1 cell, Colonic cell suspension	[179]
**Pramipexole**	Sepsis	↑ Survival rate in septic *pink1^-/-^* and *park2^-/-^* mice ↓ Inflammasome-dependent cytokines (IL-1β and HMGB1) in septic *pink1^-/-^* and *park2^-/-^* mice	↑ Circulating dopamine levels in septic *pink1^-/-^* and *park2^-/-^* mice ↓ Circulating lactate levels in septic *pink1^-/-^* and *park2^-/-^* mice ↓ Sepsis-induced *Hif1α, Ldha*, and *Pdk1* as well as GSH depletion in the brain from *pink1^-/-^* and *park2^-/-^* mice ↓ *Hif1α*-mediated aerobic glycolysis involved in inflammasome activation in *pink1^-/-^* and *park2^-/-^* mice	CLP sepsis in *pink1^-/-^*, *park2^-/-^*, *nlrp3^-/-^, hif1α^-/-^* mice	[167]
**Andrographolide**	Colitis-associated cancer	↓ Tumorigenesis and inflammation in a colitis-associated colorectal cancer model ↓ Progression of DSS-induced experimental colitis in mice	↓ NLRP3 inflammasome activation in LPS-primed macrophages treated with ATP or in mice with DSS-induced colitis ↓ ATP-induced collapse of the mitochondria membrane potential and mitochondria fragmentation in LPS-primed macrophages ↑ Mitophagy induction in macrophages	THP-1 cell, Peritoneal macrophages, BMDM, In vivo CAC mice models	[180]
**Resveratrol**	Peritoneal mesothelial cells inflammatory injury	↑ Protection from ROS-NLRP3-mediated inflammatory injury	↑ Mitophagy/autophagy via AMPK activation	Human peritoneal mesothelial cell line (HMrSV5)	[181]
**Honokiol**	Behavioral and cognitive impairment	↑ Cognitive recovery	↑ Mitophagy ↓ mtROS NLRP3 and microglia activation in the hippocampus ↓ Neuronal apoptosis	Surgery/anesthesia (sevoflurane) induced cognitive impairment in mice	[182]

PBMC: Peripheral blood mononuclear cell; AMPK: AMP-activated protein kinase; NLRP3: NLR Family Pyrin Domain Containing 3; PINK1: PTEN Induced Kinase 1; MFN2: Mitofusin 2; LPS: Lipopolysaccharide; ROS: Reactive oxygen species; BMDM: Bone marrow-derived macrophages; HepG2: Human hepatocellular carcinoma cell line; PA: Palmitic acid; OPTN: Optineurin; NIX: NIP3-like protein X; ox-LDL: oxidized low-density lipoprotein; CCH: Chronic cerebral hypoperfusion; BCCAo: Bilateral common carotid artery occlusion; AAV: Adeno-associated virus; MCAO: middle cerebral artery occlusion-reperfusion; SAH: Subarachnoid hemorrhage; Atg: Autophagy-related gene; SESN2: Sestrin 2; CLP: Cecal ligation and puncture; SQSTM1: sequestosome 1; ULK1: Unc-51 like kinase 1; UC: Ulcerative colitis; HMGB1: High mobility group box 1; DSS: Dextran sulfate sodium; Andro: Andrographolide; CAC: Colitis-associated cancer; mtROS: mitochondrial reactive oxygen species; ↑, increase; ↓, decrease.

## Abbreviations

AIM2	Absent in melanoma 2
AMPK	AMP-activated protein kinase
AMPK	5′-AMP-activated protein kinase
ASC	Apoptosis-associated speck-like protein containing a CARD
Atg	Autophagy-related gene
BCCAo	Bilateral common carotid artery occlusion
BMDM	Bone marrow-derived macrophages
CAC	Colitis-associated cancer
CCH	Chronic cerebral hypoperfusion
CK2	Casein kinase 2
CLP	Cecal ligation and puncture
DAMP	Damage associated molecular pattern
dsDNA	Double-stranded DNA
DSS	Dextran sulfate sodium
eIF2α	Eukaryotic initiation factor 2
FUNDC1	FUN14 domain containing 1
GABARAP	Gamma-aminobutyric acid receptor-associated protein
GLP-1	Glucagon like peptide-1
HepG2	Human hepatocellular carcinoma cell line
HMGB1	High mobility group box 1
IL	Interleukin
IMM	Inner mitochondrial membrane
LPS	Lipopolysaccharide
MFN2	Mitofusin 2
mtDNA	Mitochondrial DNA
mTOR	Mammalian target of rapamycin
mtROS	Mitochondrial reactive oxygen species
NAIP	NLR family, apoptosis inhibitory protein
NEK7	NIMA related kinase 7
NF-κB	Nuclear factor kappa-light-chain-enhancer of activated B cells
NIX	NIP3-like protein X
NLRC4	NLR family CARD domain containing 4
NLRP3	NOD-, LRR- and pyrin domain-containing protein 3
NR1D1	Nuclear receptor subfamily 1, group D, member 1
OMM	Outer mitochondrial membrane
OPTN	Optineurin
ox-LDL	Oxidized low-density lipoprotein
P2X7	P2X purinoceptor 7
PA	Palmitic acid
PAMP	Pathogen associated molecular pattern
PBMC	Peripheral blood mononuclear cell
PGAM5	PGAM family member 5
PHB2	Prohibitin 2
PINK1	PTEN Induced Kinase 1
PKC	Protein kinase C
PRR	Pattern recognition receptor
RIPK2	Receptor-interacting serine/threonine-protein kinase 2
ROS	Reactive oxygen species
SAH	Subarachnoid hemorrhage
SESN2	Sestrin 2
SQSTM1	Sequestosome 1
T2DM	Type 2 diabetes mellitus
TLR	Toll-like receptor
TNF	Tumor necrosis factor
TNFR	Tumor necrosis factor receptor
UC	Ulcerative colitis
ULK1	Unc-51 like kinase 1
